# Analysis of the TiO_2_ Photoanode Process Using Intensity Modulated Photocurrent Spectroscopy and Distribution of Relaxation Times

**DOI:** 10.1021/jacs.4c17345

**Published:** 2025-02-22

**Authors:** Yohei Cho, Mengya Yang, Junyi Cui, Yue Yang, Surya Pratap Singh, Salvador Eslava, Daniele Benetti, James R Durrant, Akira Yamaguchi, Masahiro Miyauchi, Fumiaki Amano

**Affiliations:** 1 Department of Materials Science and Engineering, School of Materials and Chemical Technology, Tokyo Institute of Technology, 2-12-1 Ookayama, Meguro-ku, Tokyo 152-8552, Japan; 2 Graduate School of Advanced Science and Technology, Japan Advanced Institute of Science and Technology, 1-1 Asahidai, Nomi, Ishikawa 923-1292, Japan; 3 Department of Chemical Engineering and Centre for Processable Electronics, Imperial College London, London SW7 2AZ, United Kingdom; 4 Department of Applied Chemistry for Environment, Graduate School of Urban Environmental Sciences, Tokyo Metropolitan University, 1-1 minami-Osawa, Hachioji, Tokyo 192-0397, Japan; 5 Department of Chemistry and Centre for Processable Electronics, Imperial College London, London W12 0BZ, United Kingdom; 6 SPECIFIC IKC, College of Engineering, Swansea University, Bay Campus, Fabian Way, Swansea, Wales SA1 8EN, United Kingdom

**Keywords:** photoanode, IMPS, DRT, time-resolved analysis, water oxidation, TiO_2_

## Abstract

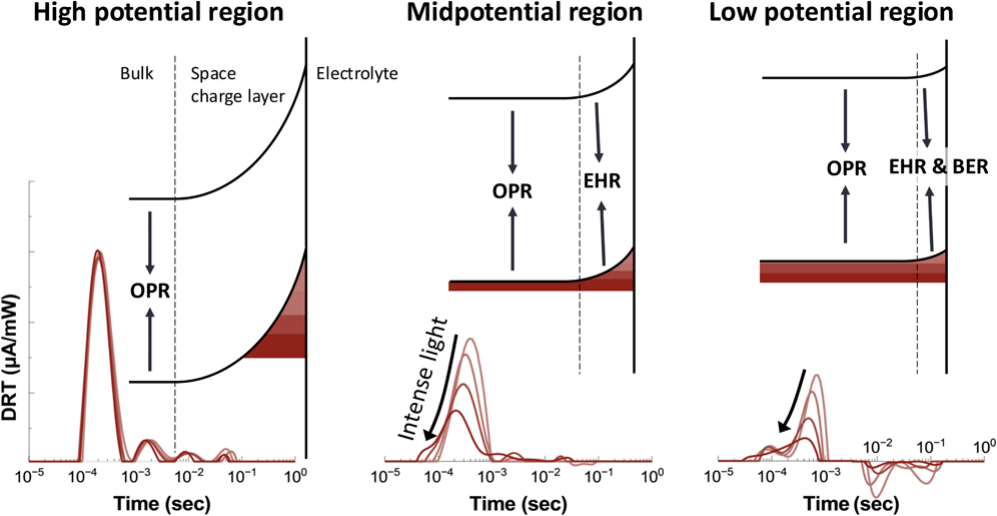

Photoelectrochemical water splitting offers a promising pathway for green hydrogen production, but its efficiency is limited by electron−hole recombination. Overcoming this challenge requires detailed analysis of the relationship between charge separation and charge transfer kinetics under operando conditions. Here, we applied intensity-modulated photocurrent spectroscopy (IMPS) combined with distribution of relaxation times (DRT) analysis to the photoanodic process under varying light intensities. This approach revealed three distinct applied potential regions: a high-potential region with constant admittance independent of light intensity; a midpotential region strongly influenced by light intensity; and a low-potential region with back electron−hole recombination (BER). Crucially, our analysis demonstrated that what has traditionally been viewed as a single bulk recombination process can be resolved into distinct mechanisms based on light intensity dependence. Additionally, we identified satellite peaks in the slow kinetic regions for the first time. These peaks, influenced by light intensity and reaction conditions, revealed novel insights into surface-trapped hole dynamics. Based on these insights, we propose tailored band bending models for each kinetic scenario and discuss the implications of satellite peaks for reaction bottlenecks. These results offer new perspectives on understanding and optimizing photoelectrochemical systems.

## Introduction

Semiconductor photoelectrochemistry is attractive as it drives uphill reactions such as water splitting^1−5^ and CO_2_ reduction.^6−9^ However, as widely recognized, due to the severe recombination of photogenerated carriers, electrons and holes, it is essential for both the carrier supply and the surface reaction to achieve satisfactory quantum efficiency. If only the carrier supply is fast, the excited carrier will cause recombination either within the bulk or at the surface. Conversely, the slower carrier supply leads to carrier depletion at the surface region. Thus, it is important to compare these kinetics and build a rational strategy for photoelectrode material development. However, separating the steps of photoelectrochemical processes starting from photoexcitation to the chemical reaction has been technically challenging, and the discussion is insufficient, especially under operando conditions.

In this study, we aimed to identify the critical limitations in a photoelectrochemical process by employing intensity-modulated photocurrent spectroscopy (IMPS) and distribution of relaxation times (DRT) analysis. IMPS is a frequency-domain method to monitor photocurrent responses to light-intensity oscillations, crucial for analyzing the kinetics of semiconductor electrodes under steady-state conditions.^10−12^ Contrary to photoelectrochemical impedance spectroscopy (PEIS),^13,14^ which observes the photocurrent response to potential fluctuations, IMPS maintains a constant potential. This is advantageous because it minimizes alterations in band bending in the space charge layer (SCL). Additionally, IMPS outputs data in units of admittance, *Y*(ω) (μA/mW), which is defined by the collection of photocurrent divided by the incident photons and is easily interpretable as quantum efficiency at low frequency (ω → 0). We utilize DRT for analyzing IMPS results to bypass the need for an equivalent circuit model, which requires preliminary knowledge and the use of complex components such as the constant phase element (CPE) for frequency-dependent spectroscopy. The DRT analysis enables us to process the IMPS results without requiring prior knowledge, mathematically breaking down the frequency-resolved IMPS spectra into a time-resolved distribution of admittance.^15−18^ For the model photoelectrochemical reaction, we selected water oxidation due to its significance as a key process in various photoelectrochemical processes, particularly as the rate-limiting step in hydrogen production through water splitting. IMPS experiments were performed using n-type titanium dioxide (TiO_2_), an extensively studied photoanodic material. We studied applied potential dependence from 0.0 to +1.6 V vs reversible hydrogen electrode (V_RHE_), and UV light intensity dependence from 1.0 to 10 mW/cm^2^, which corresponds to the range TiO_2_ can absorb sunlight.

## Methods

### Photoanode Preparation

The detailed preparation method is described in the Supporting Information. Briefly, the TiO_2_ photoanode was prepared by aerosol-assisted chemical vapor deposition (AACVD). Initially, Ti_7_O_4_(OEt)_20_ clusters were prepared from Ti(OEt)_4_ in toluene through controlled hydrolysis at ambient conditions.^19^ These clusters were then dissolved in toluene (0.05 M) and aerosolized using an atomizer with nitrogen as the carrier gas, maintaining a flow rate of 1.5 L/min. The AACVD procedure was performed for 1 h at 500 °C on fluorine-doped tin oxide-coated aluminoborosilicate glass substrates (Solaronix, CH). Then, the sample was annealed at 800 °C for 2 h in air.^20^

### IMPS Experiment and DRT Analysis

All photoelectrochemical measurements were conducted using a three-electrode system with platinum as the counter electrode and Ag/AgCl as the reference electrode. The pH value was controlled to 6.8 using a 0.1 M sodium phosphate (NaPi) buffer solution for 0.2 M sodium sulfate (Na_2_SO_4_), unless otherwise noted. For comparison, an additional experiment was conducted by adding 10 wt % ethanol (EtOH). The cell temperature was kept at 50 °C using a jacket connected to a water circulation system. The reaction temperature was set to 50 °C based on previous findings showing improved reaction kinetics at this temperature and its relevance to practical conditions under sunlight exposure.^21^ Electrode potential was applied from 0.0 to +1.6 V_RHE_, and light intensity (375 nm) was controlled from 1 to 10 mW/cm^2^ with a modulation within 33% of the continuous light intensity, to minimize the noise levels at high-frequency regions. The modulation intensity did not affect the IMPS response, indicating the linear relationship between photocurrent and the light intensity in the experimental condition (Figure S1 to S3 in Supporting Information). The modulation frequency was varied from 100 kHz to 1 Hz. The DRT analysis was based on previous reports and the code uploaded to GitHub.^17^ Only the regression part was customized to Elastic Net to reflect and better fit the experimental results, and the hyperparameter α, which determines the distribution of the regularization term, was set to 0.5 (Figure S4).

## Results and Discussion

### DRT Analysis and Assignment of Peaks

**Figure 1:**
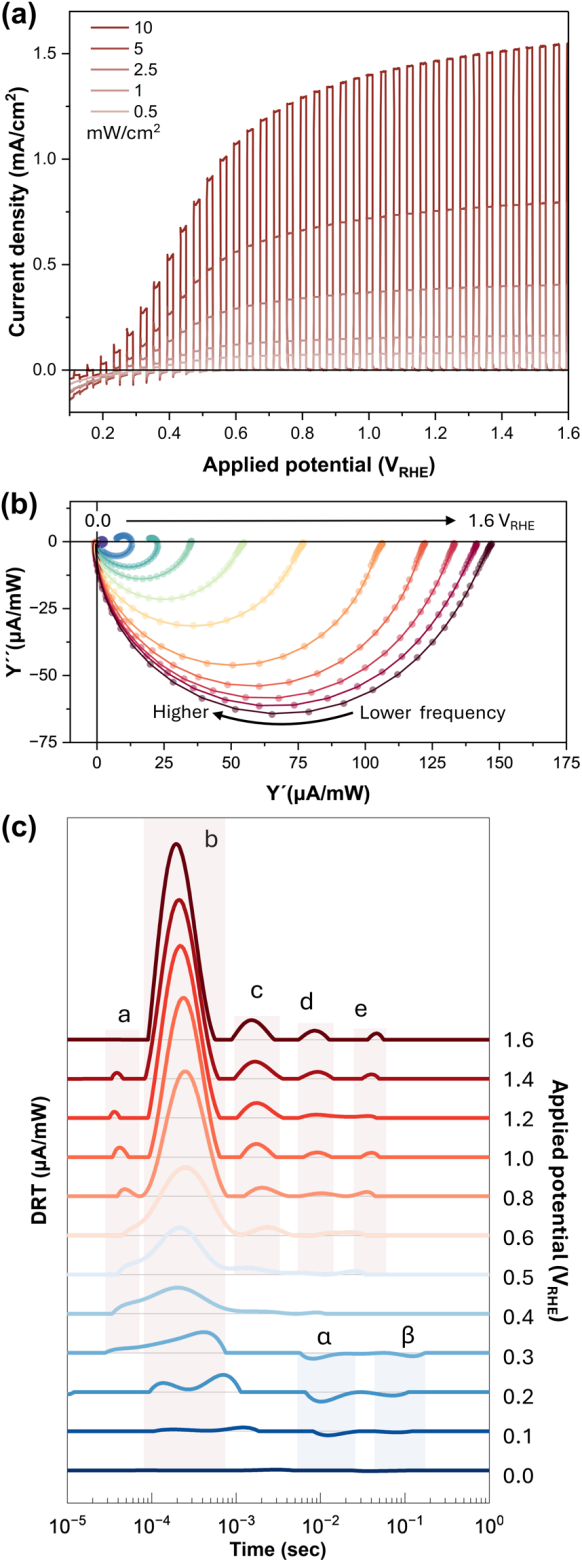
(a) Chopped linear sweep voltammetry with different UV light intensities (0.5−10 mW/cm^2^). The sweep rate is 20 mV/s, and a repeating pattern of 1 s of light exposure and 1 s of dark. (b) Nyquist plots of the IMPS response at different applied potentials (0.0−1.6 V_RHE_) under 10 mW/cm^2^ light irradiation in the frequency range of 1−100 kHz. The experimental data points are represented by dots, while the lines are the fitted curves in DRT analysis. (c) IMPS−DRT spectra derived from the fitting process. The colors in (b) and (c) represent the same applied potential.

Figure 1(a) shows the result of chopped-linear sweep voltammetry. At each light intensity, the current spikes are shown at low applied potentials right after the light turns off. This can be assigned to the back electron−hole recombination (BER), where surface holes recombine with inversely flowed electrons from the substrate.^22−24^ We converted the data in Figure 1(a) into admittance and incident photon-to-current conversion efficiency (IPCE) (Figures S5 and S6). The IPCE values at 375 nm were ∼50% at +1.2 V_RHE_.

In Figure 1(b), Nyquist complex plane plots obtained from the IMPS response at 10 mW/cm^2^ are presented as dot plots. It can be observed only at low applied potentials, the Nyquist plot extends into the first quadrant at lower frequencies, which shows consistent behavior with the previous results of α-Fe_2_O_3_ and BiVO_4_ photoanodes.^18,25−28^ This first quadrant part corresponds to BER, and is consistent with the cathodic current spikes observed at low potentials in Figure 1(a). At high potentials, the entire Nyquist plot is contained within the fourth quadrant.^29^ It can also be observed that the Nyquist plots have distorted shapes rather than a perfect semicircle, which implies the need to use CPE if we perform equivalent circuit analysis.

DRT analysis, on the other hand, allows us to directly represent this deviation from a semicircle as a time distribution. The fitted curves obtained in the DRT analysis were overlapped with the experimental data in Figure 1(b), which shows good fitting. The corresponding DRT curves are presented in Figure 1(c) as a function of relaxation time. In this figure, seven series of peaks were observed consisting of five positive peaks **a**−**e** and two negative peaks **α** and **β**. A small peak appearing early, labeled as **a**, is probably due to noise in IMPS response at high frequency and will not be discussed in this paper. The most significant positive peak, denoted as peak **b** (referred to as the “main peak” hereafter), is located in the submillisecond time domain. The main peak corresponds to the one main semicircle observed in the fourth quadrant of the Nyquist plot (Figure 1b). When compared to the IMPS−DRT results of α-Fe_2_O_3,_^17^ the main peak **b** of TiO_2_ appeared approximately 100 times faster time constant. On the other hand, the negative peaks **α** and **β**, observed only at low potentials, exhibited similar time constants to α-Fe_2_O_3_ at low frequencies.^30,31^ Additionally, three positive satellite peaks, labeled **c**, **d** and **e,** were observed for the first time in this study, to be discussed later. These features are not clearly observed in the corresponding Nyquist plots since they are convoluted with the main semicircle at low frequencies. All Nyquist plots and DRT spectra of the IMPS experiments are provided in the Supporting Information (Figures S7 to S9).

As previously reported, positive values in DRT spectra correspond to the Gärtner current, which indicates the flux of the photogenerated hole through the SCL toward the surface.^17,32^ The Gärtner current is given by the drift current inside the SCL and the diffusion current of the hole generated in the bulk outside the SCL. Conversely, negative peaks **α** and **β** suggest the presence of a reverse current due to BER at low potentials. The total photocurrent is expressed by the sum of the Gärtner current through the SCL and the BER current at the surface.

**Figure 2:**
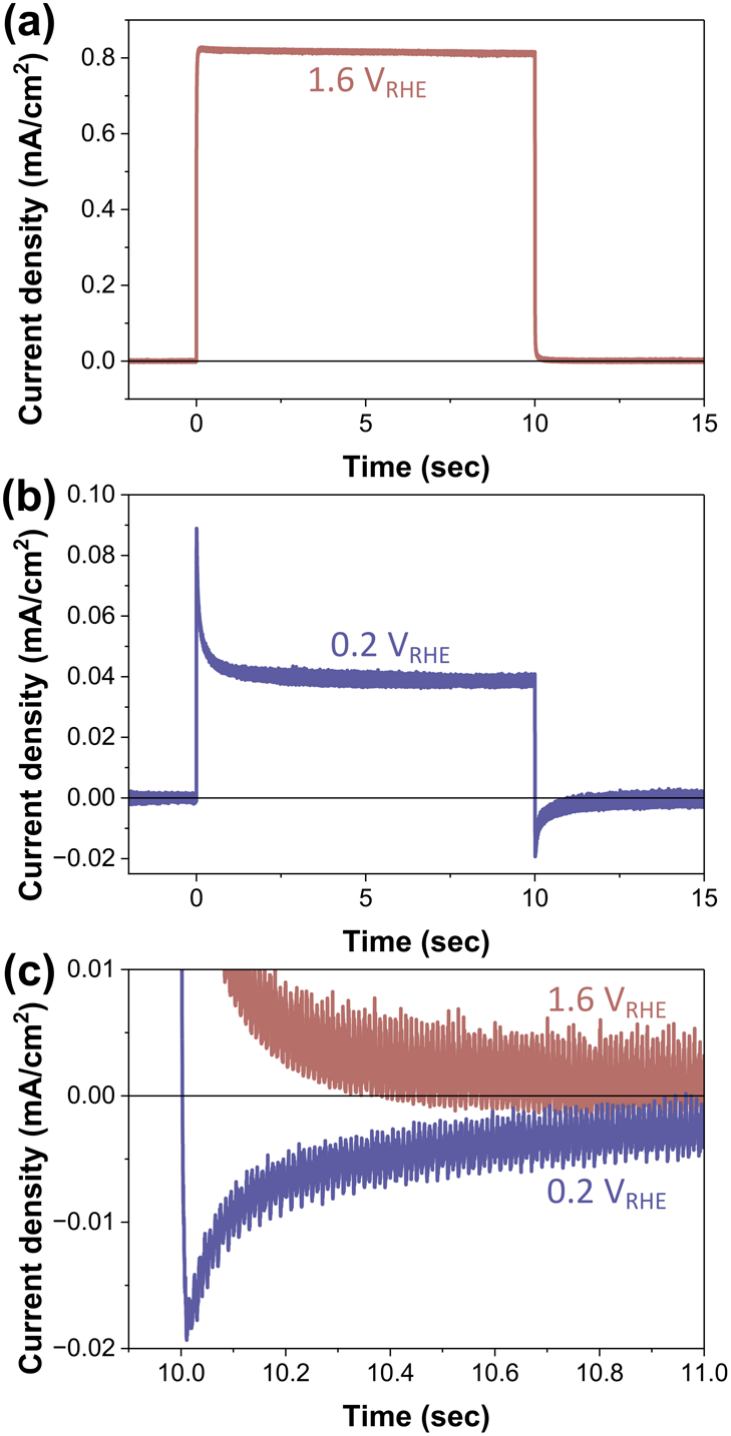
Current transient through 10-s light irradiation in chronoamperometry at (a) + 1.6 V_RHE_ and (b) + 0.2 V_RHE_. (c) The enlarged turn-off current transients at the two different potentials. The spectra were obtained under 5 mW/cm^2^ light irradiation.

We additionally discuss the peak assignment based on the time constants of photocurrent transient measurements as shown in Figure 2. In the constant light irradiation experiment, 365 nm LED was used, and light was irradiated for 10 s. The current transient behaviors at +1.6 and +0.2 V_RHE_ are shown in Figure 2(a) and (b), respectively. The magnified turn-off current transients are shown in Figure 2(c). Here, the negative transient current, which is observable only at low potentials such as +0.2 V_RHE_, appears on a time scale of hundreds of milliseconds, aligning well with the negative peak **β** in the DRT profile. The negative peak **α**, which appears earlier than **β**, could only be resolved in frequency-based experiments, but not distinguishable in conventional light-on/off experiments.

### Light Intensity Dependencies of the DRT Profile and Inferred Recombination Mechanisms

**Figure 3:**
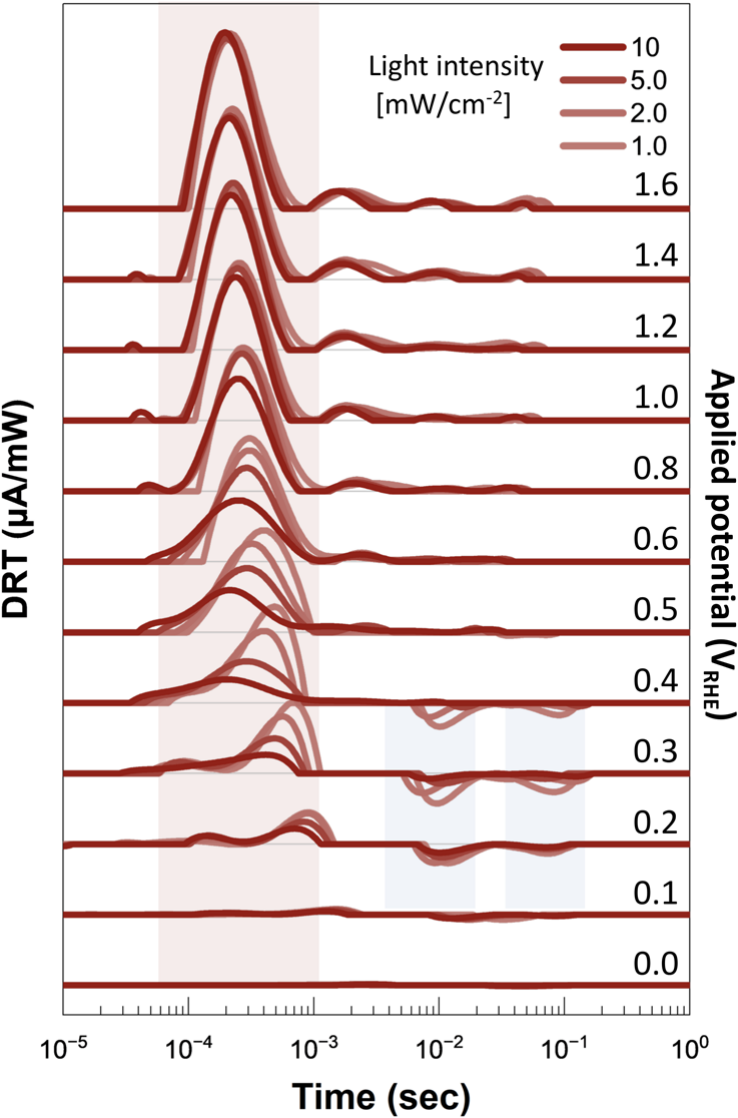
IMPS−DRT spectra obtained under UV irradiation with various light intensities (1.0−10 mW/cm^2^) at different applied potentials. The region where peak **b** is observed is shaded red, while the regions with peaks **α** and **β** are shaded blue.

Figure 3 shows the light intensity dependence of the DRT spectra. Notably, above +0.8 V_RHE_, the spectra showed no significant variation with light intensity whereas the main peak **b** exhibited clear light intensity dependence at lower potentials. It is also observable that the BER peaks **α** and **β** appears below +0.5 V_RHE_. Based on this, we categorized the applied potential region into three segments: (1) at high applied potential (>0.8 V_RHE_), DRT profile shows no light intensity dependence, (2) at 0.5−0.8 V_RHE_, light intensity dependence exists, but no BER peaks are observed, (3) at low potentials (<0.5 V_RHE_), both light intensity dependence and BER are evident.

**Figure 4:**
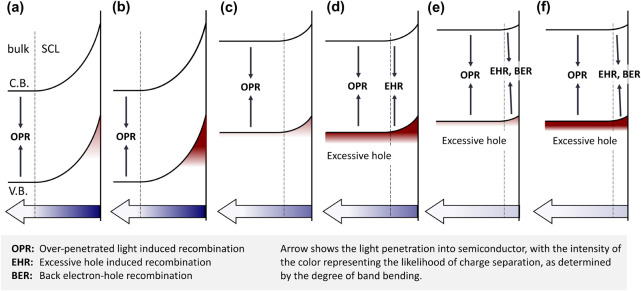
Models derived from DRT spectra showing hole densities and recombination processes on photocatalyst surfaces under various conditions: high potential (>0.8 V_RHE_) with (a) weak and (b) strong light, medium potential (0.5−0.8 V_RHE_) with (c) weak and (d) strong light, and low potential (<0.5 V_RHE_) with (e) weak and (f) strong light. Red color darkness reflects hole density, while blue color darkness indicates charge separation probability. Charge separation is evident at high potentials across all light intensities, showing no light intensity dependence. However, at midpotentials, strong light induces increased EHR in the bulk through surface hole overflow.

In the high potential region (>+0.8 V_RHE_), DRT profiles exhibit no light intensity dependence, indicating that the hole separation in the photoanode is unaffected by the photogenerated carrier density under these conditions (Figure 4(a)(b)). Upon irradiation, the first portion of the light is absorbed in the SCL, where photogenerated holes migrate to the surface driven by the band bending under high potential. The proportion of light absorbed within and beyond the SCL remains constant, regardless of the incident light intensity, as predicted by Lambert−Beer’s law, which states that the fraction of light absorbed in a material depends exponentially on the thickness and the absorption coefficient but is independent of the initial light intensity. The light penetrating beyond the SCL generates carriers deeper in the bulk, where these carriers predominantly recombine due to the absence of a built-in electric field.

At potentials below +0.8 V_RHE_, we observe a different behavior. The *area* of the main peak varies with light intensity. Here, the *area* is represented as the summation of the admittance values across a certain time range rather than the integration. The DRT spectra show a significant decrease in admittance under high light-intensity conditions, when the potential is insufficient (Figure 3). This indicates that intense light irradiation fails to adequately separate the excited carriers, decreasing the admittance at medium potentials as shown in Figure 4(c)(d). As the potential decreases, the width of the SCL narrows, and the band bending gradient becomes less pronounced, increasing the possibility of hole recombination. This leads to recombination beyond that observed at higher potentials, reducing the number of holes reaching the surface.

These observations lead us to propose a distinction between two separate recombination mechanisms within what has traditionally been treated as a single bulk recombination process.^33−35^ In the high potential region, we define Over-Penetrated light-induced Recombination (OPR), where recombination occurs due to overpenetration of light beyond the SCL. In contrast, at lower potentials with strong light irradiation, we identify Excess Hole-induced Recombination (EHR), where excess photogenerated holes favor recombination over charge separation due to reduced band bending and a narrower SCL. The key feature is that OPR occurs independently of potential, while EHR depends on the potential. These findings reveal a key novelty in our analysis: what has traditionally been described as a single bulk recombination process can now be clearly separated into two distinct mechanisms, OPR and EHR, by examining the light intensity dependence of the DRT peaks. This distinction challenges the conventional view of bulk recombination and provides a more detailed understanding of the charge recombination dynamics in photoanodes.

To discuss the excess of the hole, we measured the surface hole density through in situ photoinduced absorption spectroscopy using a UV LED pulse (Figure S10). The nonlinearity of surface hole density at 500 nm against the UV light intensity indicates more bulk recombination under stronger light illuminations. Although bulk recombination occurs even at 1.6 V_RHE_, the extent of the recombination is much more pronounced at 0.4 V_RHE_ close to the flatband potential. When the applied potentials are more positive than the flatband potential, electrons are depleted from the semiconductor to make the SCL. The width of the SCL depends on the donor density, and the potential drop distributions in the SCL are depicted in Figure S11 based on Poisson’s equation.^36^

At applied potentials lower than +0.4 V_RHE_, close to the flatband potential, in addition to the appearance of light intensity dependence, negative peaks appear in the DRT curve showing that BER occurs in this region (Figure 3). Under these conditions, the bottleneck in the photoanodic performance cannot be determined to one, and improvement is required for every process including the suppression of BER at the surface (Figure 4(e)(f)). However, discussing the extent of light intensity dependency and the areas of positive and negative peaks provides effective guidance for enhancing the photoelectrode performance.

Based on the three recombination mechanisms, i.e., OPR, EHR, and BER, we propose guidelines for developing highly efficient photocatalysts/photoelectrodes in each potential region, as shown in Table 1. These guidelines focus on minimizing the amount of each type of recombination. It should be noted that the guidance can be applied to photoelectrode materials of thin films and also photocatalytic powders if these can be deposited on a conductive substrate for IMPS−DRT analysis.

### Rate Constant Model Analysis and Kinetic Constants

**Figure 5:**
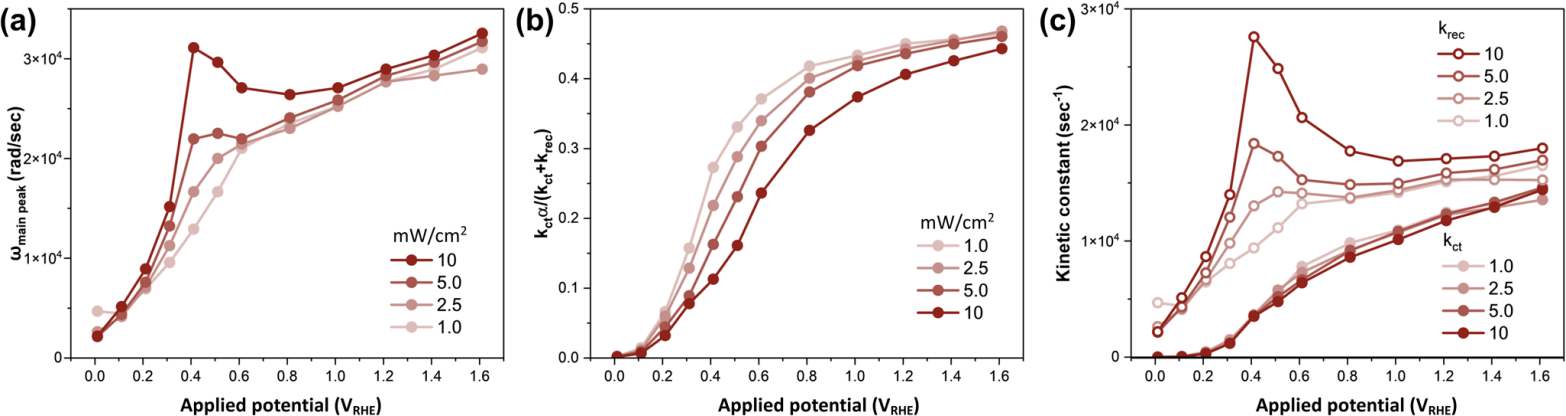
Rate constant model analysis results. (a) Angular frequency corresponding to main peak time (τ_main_) extracted from the DRT profile in Figure 3. (b) Quantum yield attributed to the main peak area, *Y*(τ_main_). (c) Kinetic parameters calculated from (a) and (b). In the graphs, the darker the line color shows the stronger the light intensity data.

For the quantitative discussions regarding the hole supply and bulk recombination kinetics, rate constant model analysis was performed. The two phenomenological rate constants, *k*_ct_ and *k*_rec_, were calculated following the previous study assuming Equation S6.^12^Figures 5(a) and (b) show the main peak position (τ_main_ = 1/ω_main_) and the *area* (*Y*(τ_main_)) in the IMPS-DRT spectra (Figure 3). Figure 5(c) shows the rate constants calculated from Figures 5(a) and (b) based on Equations S7 and S8. The rate constants, *k*_ct_ and *k*_rec_, behave differently against applied potential and irradiated light intensity. The rate constant of charge transfer, *k*_ct_, increased monotonically with an increase in the applied potential. There was no light intensity dependence in the charge transfer process. In contrast, the rate constant of recombination, *k*_rec_, demonstrated a characteristic dependence on light intensity. When the light is weak, the *k*_rec_ increased almost monotonically as the applied potential increased up to 0.6 V_RHE_, suggesting the increase in the surface hole density enhances the recombination through EHR. Stronger light intensity also increased the *k*_rec_, implying that higher photogenerated carrier density enhanced the recombination at the surface and bulk through EHR. The *k*_rec_ was maximized at +0.4 V_RHE_ under strong light (10 mW/cm^2^) since the EHR is dominant at the low potential. The higher applied potential decreases the EHR owing to the sufficient SCL width. The *k*_rec_ at higher potentials would be mainly attributed to OPR as explained in Figure 4(a) and (b), where there is no light intensity dependence.

**Table 1:** Guidance for Material Development in Each Condition

Applied potential region	Observed phenomenon	Recombination mechanism	Guidance for material development
High (>0.8 V_RHE_)	DRT spectra are light-intensity independent.	OPR	Improve light absorption more at the SCL to prohibit light penetration into the bulk.
			Increase the ratio of SCL to volume, by decreasing semiconductor size with suitable carrier density.
Medium (0.5−0.8 V_RHE_)	The main peak of the DRT spectra shows the light-intensity dependence.	Under weak light: OPR	Under weak light: The same strategy as in the high-potential region should be applied.
		Under strong light: OPR and EHR	Under strong light: As surface hole density is saturated, enhance the surface reaction rate to decrease EHR.
Low (<0.5 V_RHE_)	Both the main peak and BER exhibit light-intensity dependence.	OPR, EHR and BER	Decrease both BER and EHR. Looking at the light-intensity dependence will provide the information that affects more.

The main peak position shift in Figure 3, as plotted in Figure 5a, can be explained by the combined effects of *k*_ct_ and *k*_rec_. According to eq S10, the peak position, τ_main_, is determined by the inverse of the sum of *k*_ct_ and *k*_rec_. At lower potentials where potential-dependent EHR occurs, both *k*_ct_ and *k*_rec_ increase with the applied potential, leading to a shift in the peak position. However, in the high-potential region where EHR is absent, the peak position is primarily influenced by *k*_ct_ alone. This can be attributed to the stronger band bending at higher potentials, which enhances carrier transport and the subsequent charge transfer process.

We have also performed similar experiments in the presence of ethanol as a sacrificial electron donor (Figure S12). The DRT peaks with ethanol exhibited no negative BER peak, a shift to a slower time scale, and an increase in the *area* in the main peak. The suppression of BER at lower potentials suggests that the ethanol inhibited the surface recombination. The time shift and enhanced *area* (photocurrent density) were significant only at lower potentials. This trend can be understood by rate constant analysis, as shown in Figure S13, where the addition of ethanol does not affect *k*_ct_ while it significantly affects the *k*_rec_, especially at lower applied potentials. Interestingly, there was no significant difference in the charge transfer kinetics on the TiO_2_ photoanode for water oxidation and ethanol oxidation. The *k*_rec_ is defined to be either OPR or EHR, and bulk OPR should not be affected by the ethanol addition. Therefore, the less *k*_rec_ with ethanol suggests that EHR would be suppressed by the decrease in the surface hole density.

### Discussions Regarding the Satellite Peaks

**Figure 6:**
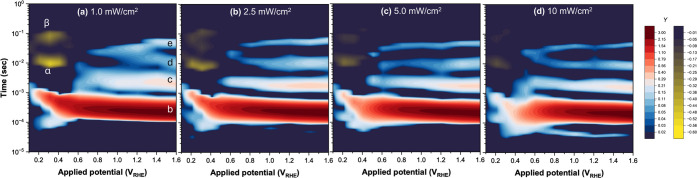
IMPS−DRT maps presented with the applied potentials under different light intensities: (a) 1.0, (b) 2.5, (c) 5.0, and (d) 10 mW/cm^2^, respectively. Positive peaks in the DRT function are represented by a color gradient from orange to dark red, while negative peaks correspond to the color gradient from green to dark blue.

Now, we turn to the discussion of satellite peaks **c**, **d**, and **e**. It should first be noted that these satellite peaks have not been reported previously and are observed here for the first time. One initial observation is that these peaks influence the real part as ω approaches 0, contributing to the steady-state photocurrent in Nyquist plot. Figure 6 presents the IMPS−DRT maps under different light intensities and applied potentials. By examining this figure, along with Figure S9, it becomes evident that there is no potential where both the BER and the satellite peaks coexist. This lack of coexistence at a slower time scale (10^−3^∼10^−1^ second) suggests that the surface-trapped holes may be directed to either process depending on the conditions. When the applied potential is insufficient (<+0.5 V_RHE_), the excited electrons and holes remain close to each other, leading to BER. However, as the applied potential becomes more anodic (>+0.5 V_RHE_), BER is suppressed due to increased spatial separation of excited electrons into the bulk.

Focusing on the surface reactions, the presence of ethanol suppresses BER, causing the first quadrant to disappear (Figure S14). This is explained by the holes contributing to charge transfer rather than BER, ethanol oxidation is faster than water oxidation. Even near the photocurrent onset potential for water oxidation, ethanol oxidation occurred by the surface-trapped holes. The presence of ethanol also distorted the semicircle in the fourth quadrant, indicating heterogeneity in the surface reaction kinetics. The satellite peaks were further enhanced during ethanol oxidation.

To discuss satellite peaks, we focus on the effect of changing light intensity on the normalized Nyquist plots (Figure S8). At lower potentials, such as 0.3−0.4 V_RHE_, a distorted semicircle in the first quadrant was observed at lower frequencies. The semicircle diameter, which corresponds to the BER peak area (Figure 3), decreases as the light intensity increases, suggesting that higher surface hole density decreases the BER probably due to the enhanced surface reaction. At middle potentials around 0.5 V_RHE_, the slope angle at which the Nyquist plot intersects the real axis on the low-frequency side (the right side of the graph) decreases from ∼90° to ∼45° as the light intensity increases, indicating that the photocurrent densities are gradually increased in the slow time domain (Figure S8). This slow reaction dynamics contributes to the satellite peaks in IMPS−DRT spectra (Figure 3). In contrast, at high potentials above 1.0 V_RHE_, the satellite peaks are less dependent on the light intensity. These results suggest that the increased surface hole density facilitates surface reactions rather than the BER.

The specific origin of the satellite peaks remains unclear from IMPS-DRT alone, reflecting the complexity of interfacial processes. While IMPS provides valuable time-resolved insights into photocurrent dynamics, it may overlook phenomena such as multihole exchanges or surface-bound intermediates. Complementary techniques, including in situ FTIR, Raman spectroscopy, pump−probe spectroscopy, and EPR, can provide additional perspectives. Integrating these methods with IMPS allows a more comprehensive understanding of photoelectrochemical reactions.

## Conclusions

We conducted IMPS measurements on water oxidation using anatase TiO_2_ as a model photoanode. The DRT analysis identified one main positive peak, three smaller positive peaks, and two negative peaks, each with different time constants. The DRT profile of the main peak could be classified into three applied potential regions: a high-potential region independent of light intensity, a midpotential region dependent on light intensity, and a low-potential region dependent on light intensity and exhibiting negative peaks. From these observations, we discussed three recombination processes; back electron−hole recombination (BER), excess hole-induced recombination (EHR), and overpenetrated light-induced recombination (OPR) occurring at each potential region that limits photoelectrochemical performance. Rate constant model analysis further confirms that EHR occurs at the midpotential region under strong light irradiation. The satellite peaks were also discussed, revealing that their dependence on potential and light intensity in charge transfer kinetics suggests they compete with BER depending on the surface hole density. This study is the first to report the presence of satellite peaks in the DRT spectra, aside from the main process, a discovery made possible only by combining IMPS and DRT analysis. We believe this discussion aids in understanding the mechanisms and bottlenecks, providing guidance for efficient photocatalytic and photoelectrochemical materials development.

## Supporting Information Available

Detailed material preparation method, IMPS experiment conditions, the optimization of modulation intensity, hyperparameter selection for Elastic Net regression in DRT analysis, raw and normalized Nyquist plots, all the obtained DRT spectra, density of surface holes measurement, potential estimation at the photoanode surface, DRT spectra with ethanol, rate constant model analysis with ethanol, Nyquist plot with ethanol (PDF)

## ABBREVIATIONS

IMPS: intensity modulated photocurrent spectroscopy
DRT: distribution of relaxation times
BER: back electron−hole recombination
PEIS: photoelectrochemical impedance spectroscopy
SCL: space charge layer
AACVD: aerosol-assisted chemical vapor deposition
OPR: overpenetrated light-induced recombination
EHR: excess hole-induced recombination.
CPE: constant phase element
