# Supplementary material for: Analysis of the TiO2 Photoanode Process Using Intensity Modulated Photocurrent Spectroscopy and Distribution of Relaxation Times
Source: J Am Chem Soc. 2025 Feb 22;147(9):7703–10. doi: 10.1021/jacs.4c17345 (PMC11887061; doi:10.1021/jacs.4c17345)
Supplement: Supplementary file 1 — ja4c17345_si_001.pdf [file ja4c17345_si_001.pdf]

Supporting Information

Analysis of TiO_2 Photoanode Process Using Intensity Modulated Photocurrent Spectroscopy and Distribution of Relaxation Times

Authors: Yohei Cho[1,2], Mengya Yang[3], Junyi Cui[3], Yue Yang[1], Surya Pratap Singh[4], Salvador Eslava[3], Daniele Benetti[5], James R Durrant[5,6], Akira Yamaguchi[1], Masahiro Miyauchi[1], and Fumiaki Amano[4*]

[1] Department of Materials Science and Engineering, School of Materials and Chemical Technology, Tokyo Institute of Technology, 2-12-1 Ookayama, Meguro-ku, Tokyo 152-8552, Japan

[2] Graduate School of Advanced Science and Technology, Japan Advanced Institute of Science and Technology, 1-1 Asahidai, Nomi, Ishikawa 923-1292, Japan

[3] Department of Chemical Engineering and Centre for Processable Electronics, Imperial College London, London SW7 2AZ, United Kingdom

[4] Department of Applied Chemistry for Environment, Graduate School of Urban Environmental Sciences, Tokyo Metropolitan University, 1-1 Minami-Osawa, Hachioji, Tokyo 192-0397, Japan

[5] Department of Chemistry and Centre for Processable Electronics, Imperial College London, London W12 0BZ, United Kingdom

[6] SPECIFIC IKC, College of Engineering, Swansea University, Bay Campus, Fabian Way, Swansea, Wales, SA1 8EN, United Kingdom

*E-mail: f.amano@tmu.ac.jp

1. Experimental methods

Materials Section

Sigma Aldrich supplied Titanium(iv) ethoxide $Ti(OEt)_4$, anhydrous toluene with a purity of over 99.9%, and ethanol containing less than 0.0003% water. Aluminoborosilicate glass substrates coated with a transparent layer of fluorine-doped tin oxide (FTO) with a resistance of 8 Ω per square were obtained from Solaronix SA in Switzerland. These substrates underwent a cleaning process involving ultrasonic treatment in a 2% Hellmanex III aqueous solution, followed by sequential rinsing with deionized water, acetone, and isopropyl alcohol, each for 3 minutes. A final rinse with deionized water and subsequent drying was performed.[1]

Preparation of TiO_2 Photoanodes

The fabrication of TiO_2 photoanodes on FTO-coated aluminoborosilicate glass involved the use of an aerosol-assisted chemical vapour deposition technique, as detailed in previous studies by Regue and colleagues.[2] The process began with the creation of $Ti_7O_4(OEt)_{20}$ clusters through careful hydrolysis of $Ti(OEt)_4$ in toluene at ambient temperature. These clusters were then dissolved in toluene to achieve a 0.05 M solution. This solution was aerosolized using a TSI Model 3076 Constant Output Atomizer, employing nitrogen as the propellant gas at a flow rate of 1.5 litres per minute. The aerosol deposition of $Ti_7O_4(OEt)_{20}$ onto the FTO-coated glass substrates was performed in a tube furnace with a 34 mm diameter, at a temperature of 500 °C for one hour. After deposition, the substrates were allowed to cool under a continuous flow of nitrogen. The final step involved annealing the films in air, by ramping up the temperature at a rate of 10 °C per minute to 800 °C and maintaining this temperature for two hours, before letting them cool naturally in the ambient air.

2. IMPS experiment

UV light-emitting diode (LED) with a central wavelength emission of 375 nm was used as a light source. The light intensity emitted by the LED was tuned to 1.0, 2.5, 5.0, and 10.0 mW/cm^2. This calibration was performed using a Zahner power potentiostat (model PP211, based in Germany), ensuring precise control over the illumination conditions employed during the experiments. To conduct IMPS measurements, a Zahner impedance analyzer (Zennium model, Germany) was utilized. The frequency range was from 10 kHz to 1 Hz.

Optimization of modulation intensity

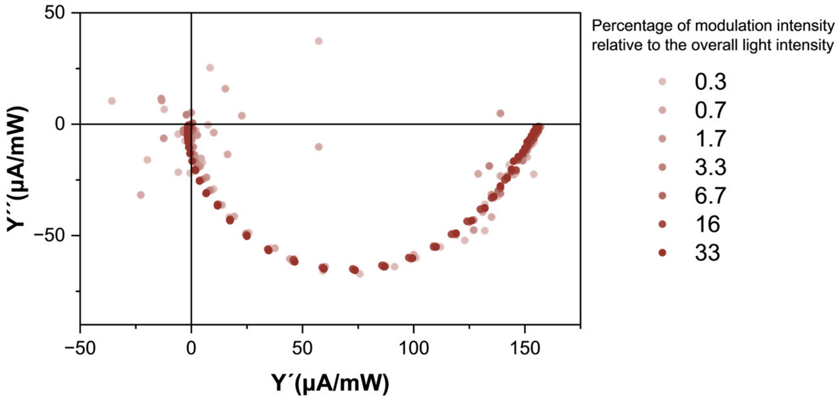

Figure S1. Modulation intensity dependence of the Nyquist plots performed at 1.6 V_{RHE}, 5 mW/cm^2 light irradiation.

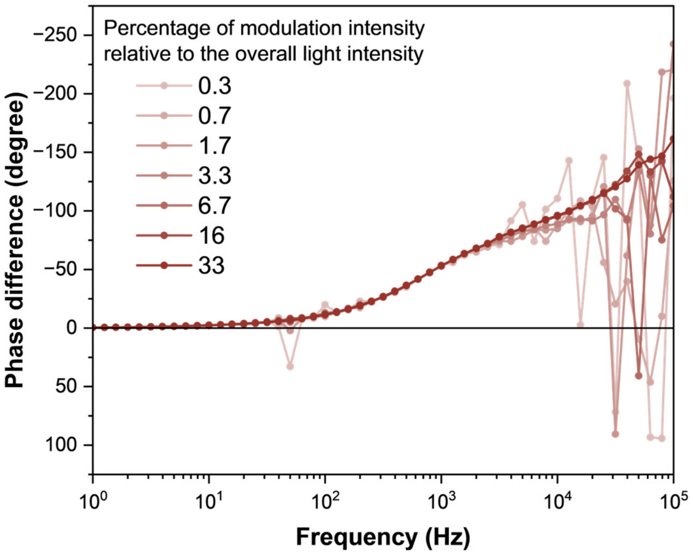

Figure S2. Modulation intensity dependence of the bode plots of the phase difference performed at 1.6 V_{RHE}, 5 mW/cm^2 light irradiation.

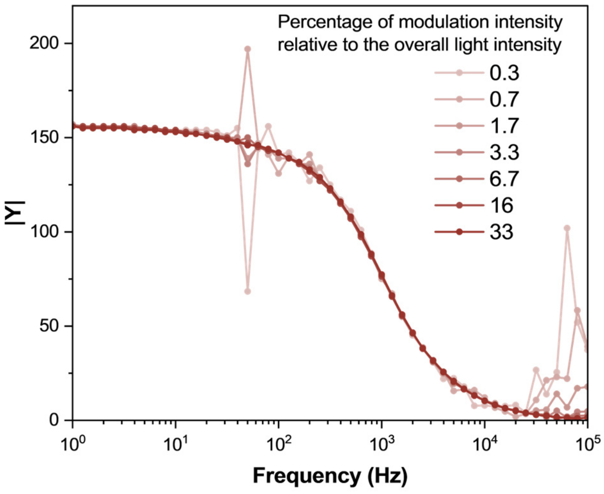

Figure S3. Modulation intensity dependence of the bode plots of the admittance performed at 1.6 V_{RHE}, 5 mW/cm^2 light irradiation.

In this study, we optimized the light intensity modulation to achieve a balance between noise reduction and signal stability. Figures S1−S3 demonstrate the results of varying the modulation from 0.3% to 33%. As seen in the figures, lower modulation intensities (e.g., 0.3%) resulted in a significant increase in noise, which hindered the detection of higher-frequency components. Conversely, when the modulation intensity was increased to 33%, we observed that the noise decreased without introducing non-linearities in the IMPS data, suggesting that the system remained in the linear response regime. This linearity was further confirmed by the consistency of the IMPS spectra across the range of modulation intensities, with no significant changes in key features such as peak positions or amplitude. Therefore, despite the relatively large modulation value, we conclude that the 33% modulation intensity ensures sufficient signal quality while maintaining linearity in the response.

DRT analysis

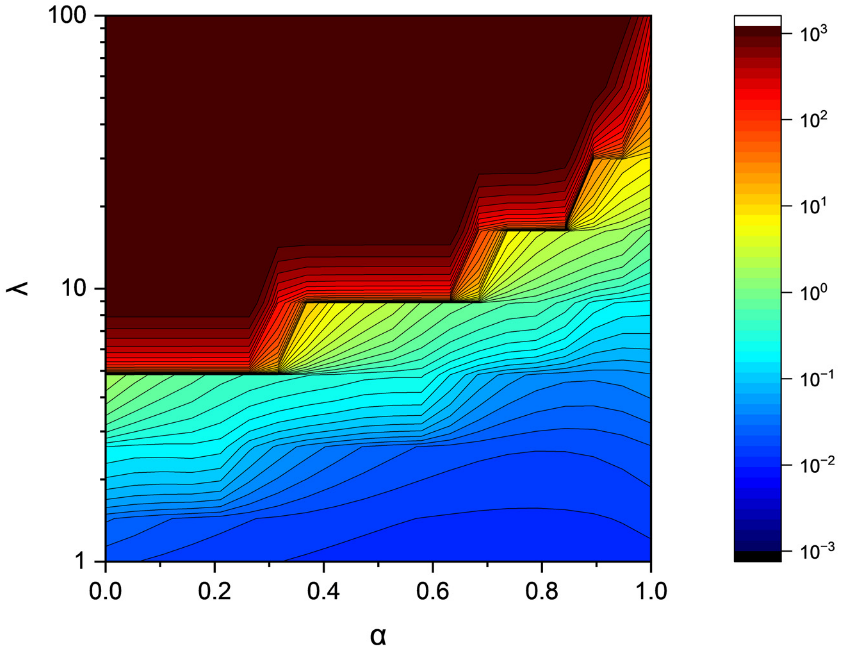

Figure S4. Grid search of the hyperparameters in Elastic Net regression for DRT analysis. The value of mean squared error (MSE) is shown as a colour variation.

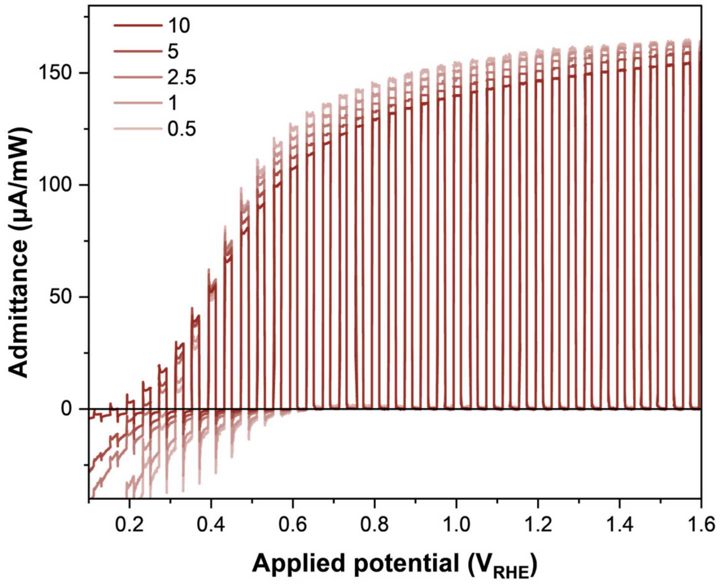

Figure S5. Admittance data of the chopped-linear sweep voltammetry shown in Figure 1(a).

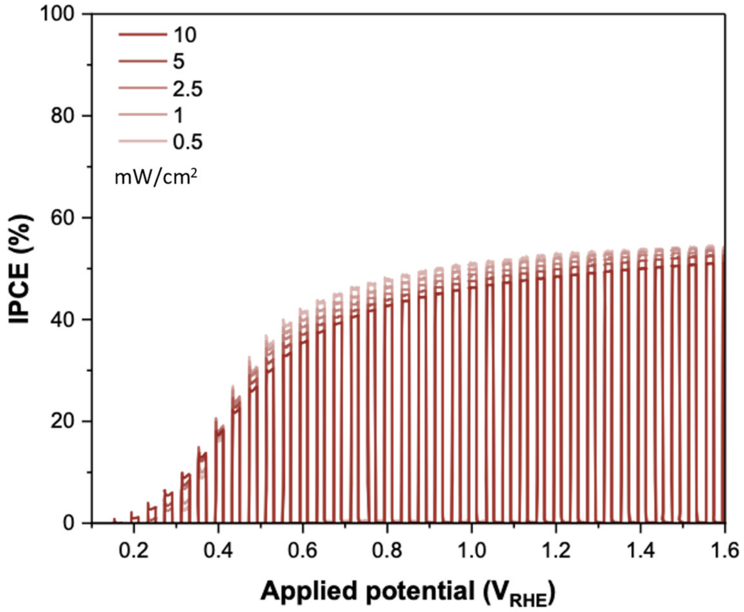

Figure S6. Incident photon to current efficiency (IPCE) data of the chopped-linear sweep voltammetry shown in Figure 1(a).

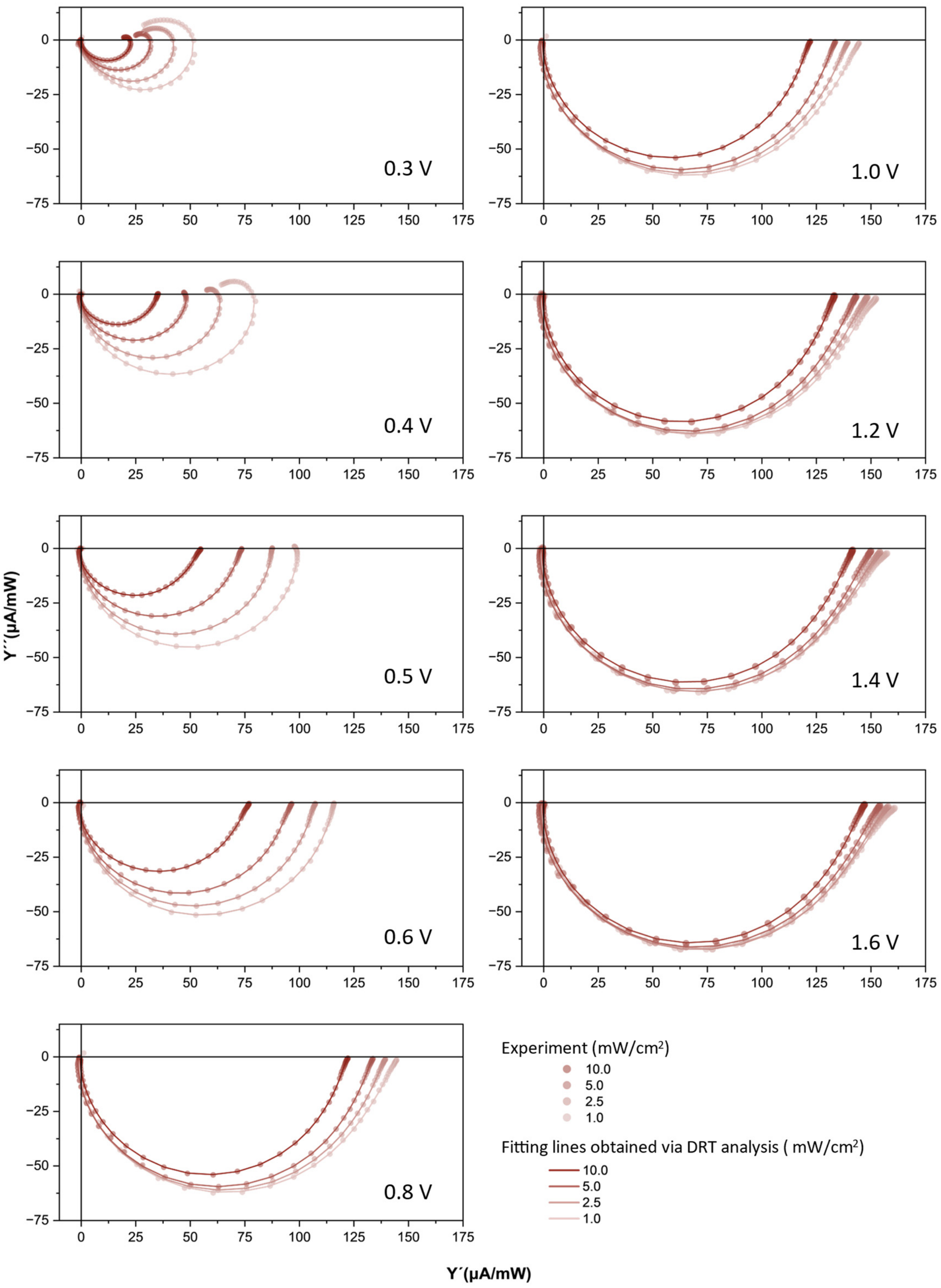

Figure S7. Nyquist plots obtained in IMPS under different incident light intensities.

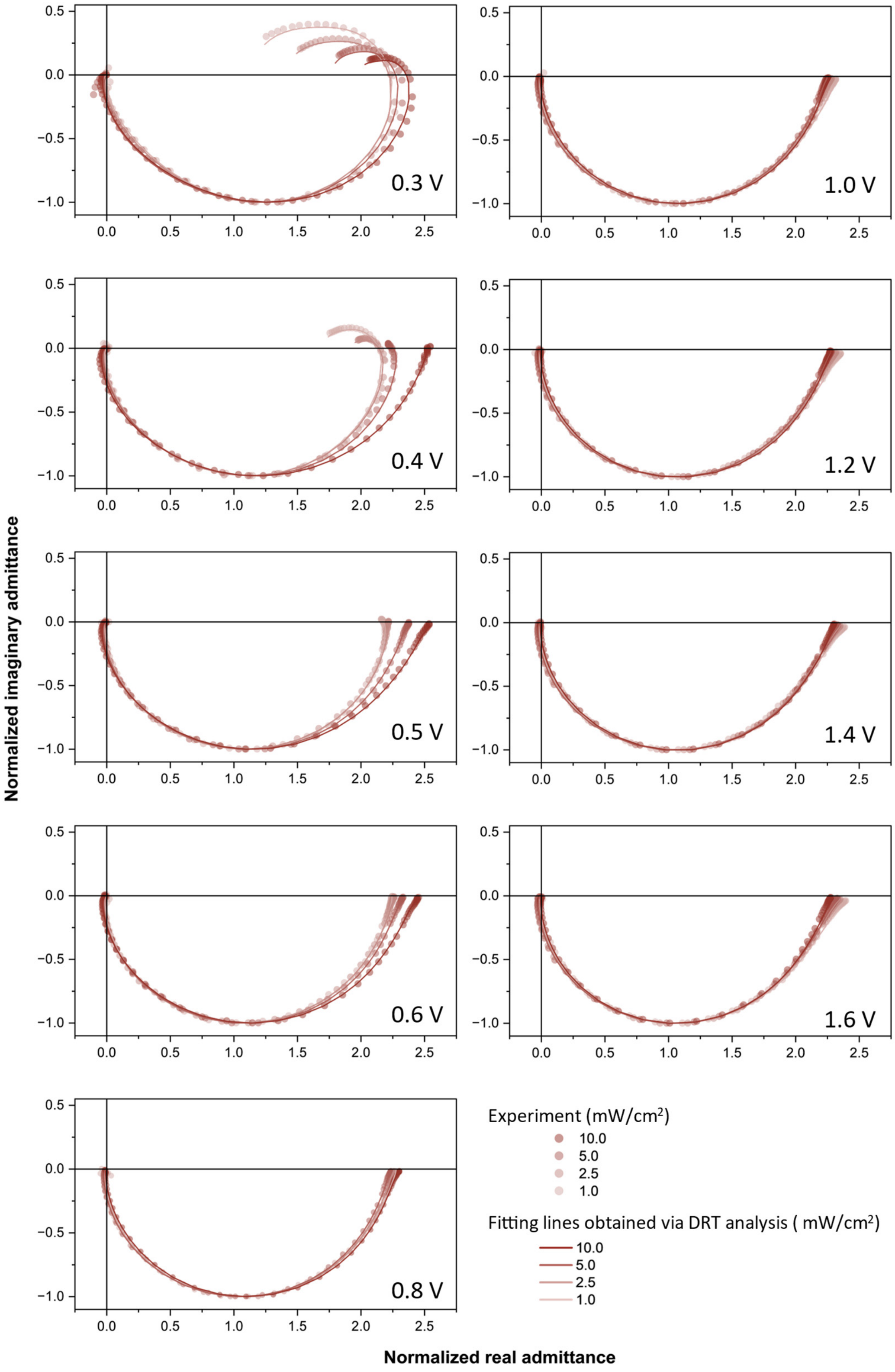

Figure S8. Normalized Nyquist plots of Figure S7. The Nyquist plots were normalized so that the minimum values on the imaginary axis were −1.0.

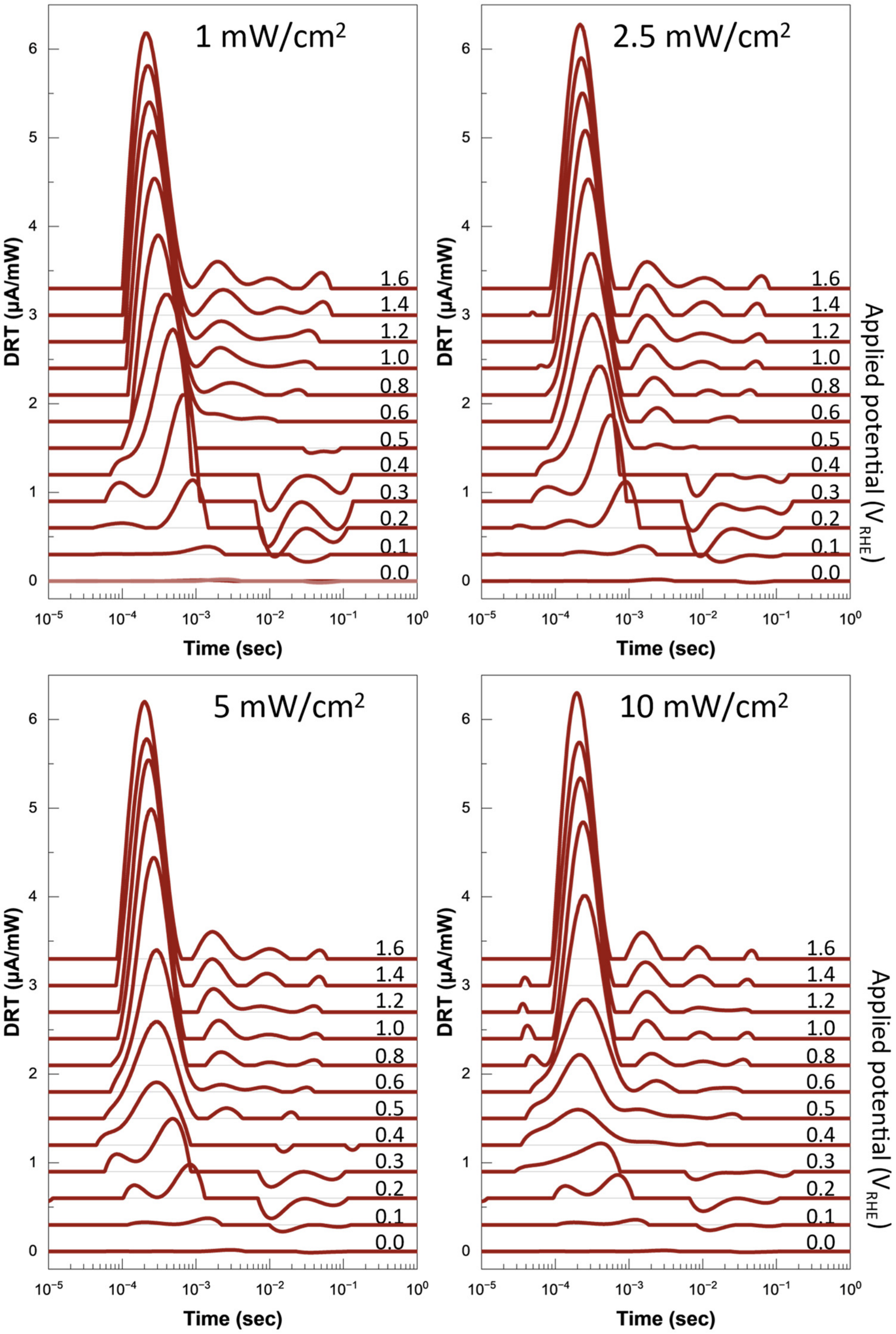

Figure S9. All the obtained DRT spectra under different light intensities: 1,0, 2.5, 5.0, and 10 mW/cm^2. The values in each figure are the applied bias (V_{RHE}).

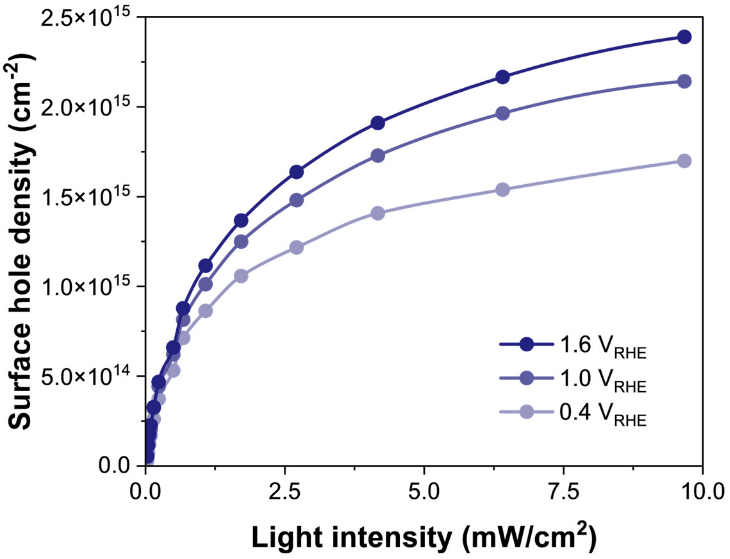

Figure S10. Density of surface holes estimated from photo-induced absorption spectroscopy (PIA) performed with 500 nm probe light and 365 nm pump light irradiation. A positive increase in the 500 nm absorption feature corresponds to an oxidation of surface states (i.e., to hole trapping). The conversion from PIA amplitude ΔO.D. to the hole number was performed following the previous literature.[3]

For the calculation of holes from the PIA result, the following equation was used. The value 5.2×10^{-8} [mol cm L^{-1}] and 0.1 mOD were obtained from the previous report.[1]

$$\text{PIA}\ [\text{m}\Delta\text{OD}] \times \frac{5.2\times10^{-8}\ \left[\frac{\text{mol}\cdot\text{cm}}{\text{L}}\right]\times 6.02\times10^{23}\ [\text{mol}^{-1}]\times10^{-3}\ \left[\frac{\text{L}}{\text{cm}^3}\right]}{0.1\ [\text{m}\Delta\text{OD}]} \qquad \text{Eq. S1}$$

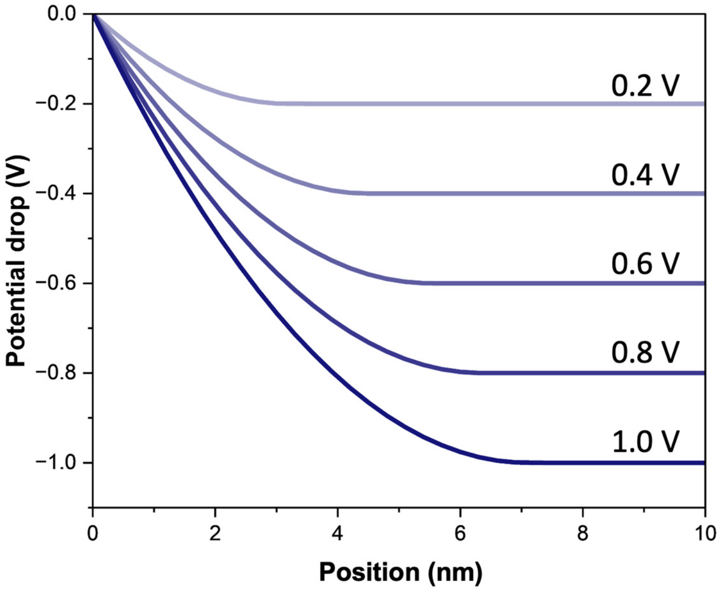

Figure S11. The potential drop calculated using the equation shown below. The donor density was obtained from Mott-Schottky analysis.

The donor density was obtained from the slope of the Mott-Schottky plot (Eq. S2).

$$N_D = \frac{2}{\varepsilon_0 \varepsilon_r q \left(\frac{d}{dV}\frac{1}{C^2}\right) S^2} \qquad \text{Eq. S2}$$

Here, we denote N_D as donor density, ε_0 as vacuum permittivity, ε_r as relative permittivity, e as elementary charge, V as potential, C as capacitance, S as surface area. We obtained $\frac{d}{dV}\left(\frac{1}{C^2}\right) = 1.7 \times 10^{10}$ from Mott-Schottky plot, and used $\varepsilon_r = 120$ as relative permittivity. As a result, $N_D = 2.2 \times 10^{10}$ was obtained.
The Eq. S3 was used to calculate the width of the space charge layer further.

$$W = \left(\frac{2\varepsilon_0 \varepsilon_r}{qN_D}\right)^{1/2} \left(V - V_{fb} - \frac{kT}{q}\right)^{1/2} \qquad \text{Eq. S3}$$

The value W is the width of the space charge layer, V_{fb} is flat-band potential, k is Boltzmann constant, and T is temperature.
The band bending of the space charge layer was further calculated by Poisson's equation (Eq. S4). Using the condition that there is no electric field at the interface, Eq. S5 is derived.

$$\frac{d^2\varphi_n}{dx^2} = -\frac{eN_D}{\varepsilon_0\varepsilon} \qquad \text{Eq. S4}$$

$$\varphi_n = -\frac{eN_D}{2\varepsilon_0\varepsilon}(x+W)^2 \qquad \text{Eq. S5}$$

The value φ_n is a potential drop at a certain depth of x from the surface.

The assumptions for rate constant model (RCM) analysis [4]

$$\frac{\mathrm{d}p}{\mathrm{d}t} = I\alpha - k_{ct}p - k_{rec}p \qquad \text{Eq. S6}$$

p: concentration of photogenerated
I: the intensity of the incident light
α: photoabsorption efficiency
k_{ct}: charge transfer rate constant
k_{rec}: recombination rate constant

Two kinetic constants are calculated using the following equation.

$$\omega_{min} = k_{rec} + k_{ct} \qquad \text{Eq. S7}$$

$$Y_{real,\omega\to 0} = \frac{k_{ct}\alpha}{k_{ct} + k_{rec}} \qquad \text{Eq. S8}$$

ω_{min}: The angular frequency where the Nyquist plot goes to the lowest point. In this paper, we used the main peak position in DRT spectra.[5] Here, the relation between angular frequency and time (as expressed in Eq. S9) was employed.

$$\omega_{min} = \frac{1}{\tau_{min}} \qquad \text{Eq. S9}$$

$Y_{real,\omega\to 0}$: Low-frequency real intercept of Nyquist plots in IMPS experiments. This value corresponds to the summation of the admittance values at the main peak, as discussed in the main paper and a previous report.

The equation can be further converted as follows, where the peak position is expressed as the inverse of the sum of k_{rec} and k_{ct}

$$\tau_{min} = \frac{1}{k_{rec} + k_{ct}} \qquad \text{Eq. S10}$$

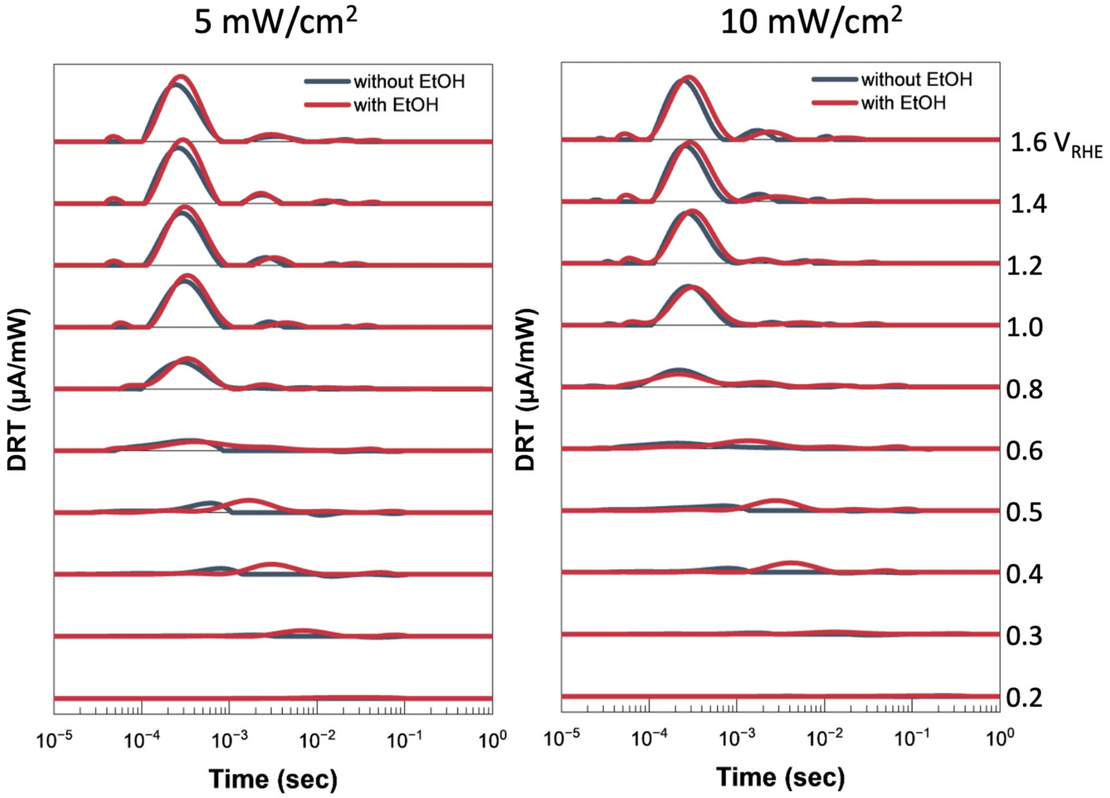

Figure S12. IMPS–DRT spectra obtained under UV irradiation at 5.0 and 10 mW/cm^2, at different applied potentials, with and without the presence of ethanol.

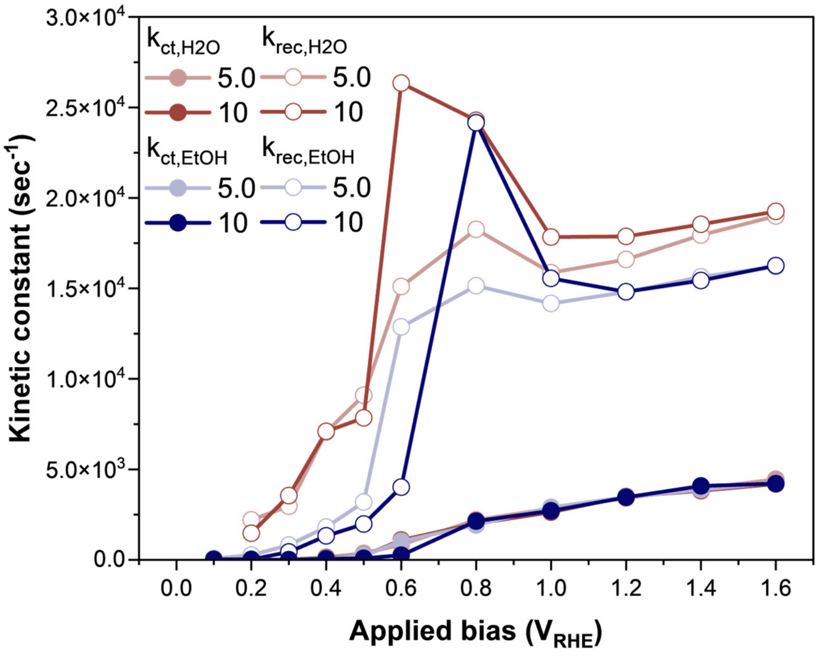

Figure S13. The kinetic constants calculated using the rate constant model (RCM) analysis. Blue represents results with ethanol, and red represents results without ethanol. Darker shades correspond to 10 mW/cm^2, while lighter shades correspond to 5 mW/cm^2. Filled symbols indicate k_{ct}, and unfilled symbols indicate k_{rec}.

Ethanol addition significantly decreased the k_{rec} at lower applied biases, resulting in the shift of the peak position of k_{rec} to higher potentials. This behavior can be explained by the faster charge transfer kinetics of ethanol oxidation, which prevents the accumulation of excess holes. The suppression of excess holes reduces EHR, leading to a decrease in the overall k_{rec}.

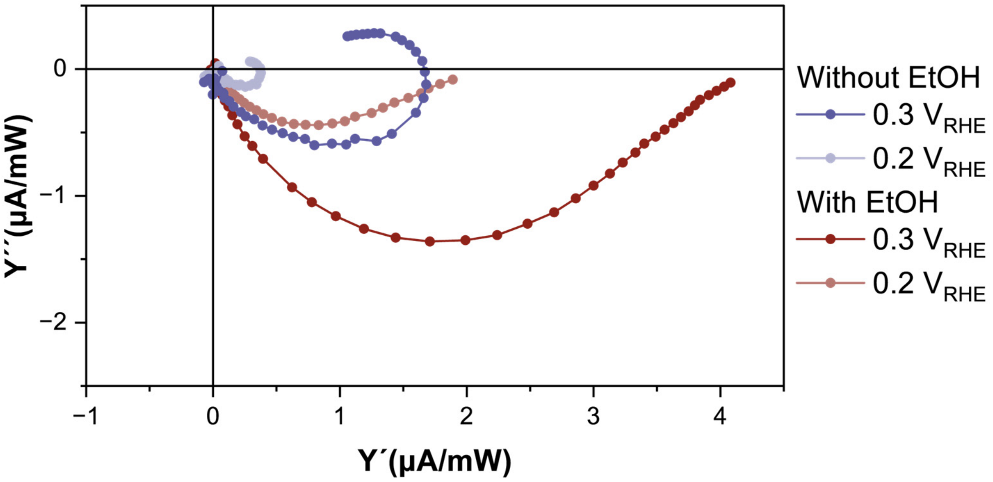

Figure S14. Nyquist plots of IMPS experiments conducted with and without ethanol at low bias under 10 mW/cm^2 light intensity.

Reference

(1) Eslava, S.; Goodwill, B. P. R.; McPartlin, M.; Wright, D. S. Extending the Family of Titanium Heterometallic–Oxo–Alkoxy Cages. *Inorg. Chem.* **2011**, *50* (12), 5655–5662. https://doi.org/10.1021/ic200350j.

(2) Regue, M.; Sibby, S.; Ahmet, I. Y.; Friedrich, D.; Abdi, F. F.; Johnson, A. L.; Eslava, S. TiO_2 Photoanodes with Exposed {010} Facets Grown by Aerosol-Assisted Chemical Vapor Deposition of a Titanium Oxo/Alkoxy Cluster. *J. Mater. Chem. A* **2019**, *7* (32), 19161–19172. https://doi.org/10.1039/C9TA04482E.

(3) Kafizas, A.; Ma, Y.; Pastor, E.; Pendlebury, S. R.; Mesa, C.; Francàs, L.; Le Formal, F.; Noor, N.; Ling, M.; Sotelo-Vazquez, C.; Carmalt, C. J.; Parkin, I. P.; Durrant, J. R. Water Oxidation Kinetics of Accumulated Holes on the Surface of a TiO_2 Photoanode: A Rate Law Analysis. *ACS Catal.* **2017**, *7* (7), 4896–4903. https://doi.org/10.1021/acscatal.7b01150.

(4) Amano, F.; Koga, S. Influence of Light Intensity on the Steady-State Kinetics in Tungsten Trioxide Particulate Photoanode Studied by Intensity-Modulated Photocurrent Spectroscopy. *J. Electroanal. Chem.* **2020**, *860*, 113891. https://doi.org/10.1016/j.jelechem.2020.113891.

(5) Piccioni, A.; Vecchi, P.; Vecchi, L.; Grandi, S.; Caramori, S.; Mazzaro, R.; Pasquini, L. Distribution of Relaxation Times Based on Lasso Regression: A Tool for High-Resolution Analysis of IMPS Data in Photoelectrochemical Systems. *J. Phys. Chem. C* **2023**, *127* (17), 7957–7964. https://doi.org/10.1021/acs.jpcc.3c00770.
